# Induced pluripotent stem cells-derived neurons from patients with Friedreich ataxia exhibit differential sensitivity to resveratrol and nicotinamide

**DOI:** 10.1038/s41598-019-49870-y

**Published:** 2019-10-10

**Authors:** Pauline Georges, Maria-Gabriela Boza-Moran, Jacqueline Gide, Georges Arielle Pêche, Benjamin Forêt, Aurélien Bayot, Pierre Rustin, Marc Peschanski, Cécile Martinat, Laetitia Aubry

**Affiliations:** 1 INSERM UMR 861, I-STEM, AFM, 91100 Corbeil-Essonnes, France; 2 UEVE, Paris-Saclay UMR 861, I-STEM, AFM, 91100 Corbeil-Essonnes, France; 3 CECS/AFM, I-STEM, 91100 Corbeil-Essonnes, France; 4 CNRS UMR 3691, Institut Pasteur, Mitochondrial Biology Group, Paris, France; 5 Hôpital Robert Debré, INSERM UMR, 1141 Paris, France

**Keywords:** Neurodegenerative diseases, Pluripotent stem cells

## Abstract

Translation of pharmacological results from *in vitro* cell testing to clinical trials is challenging. One of the causes that may underlie these discrepant results is the lack of the phenotypic or species-specific relevance of the tested cells; today, this lack of relevance may be reduced by relying on cells differentiated from human pluripotent stem cells. To analyse the benefits provided by this approach, we chose to focus on Friedreich ataxia, a neurodegenerative condition for which the recent clinical testing of two compounds was not successful. These compounds, namely, resveratrol and nicotinamide, were selected because they had been shown to stimulate the expression of frataxin in fibroblasts and lymphoblastoid cells. Our results indicated that these compounds failed to do so in iPSC-derived neurons generated from two patients with Friedreich ataxia. By comparing the effects of both molecules on different cell types that may be considered to be non-relevant for the disease, such as fibroblasts, or more relevant to the disease, such as neurons differentiated from iPSCs, a differential response was observed; this response suggests the importance of developing more predictive *in vitro* systems for drug discovery. Our results demonstrate the value of utilizing human iPSCs early in drug discovery to improve translational predictability.

## Introduction

The poor predictability of drug efficiency during clinical trials remains highly challenging because of a persistent translational gap between *in vitro* cell testing and clinical settings^1^. There is a long list of causes for such a discrepancy, including the use of tumor cells (or lymphoblastoid cell lines) with abnormal karyotypes, selection of sub-populations in cell cultures, genetic drift of the cells under study, non-physiological culture conditions, and even unrevealed contamination^2,3^. The advent of human pluripotent stem cells (hPSCs) in the pharmacological field over the past decade has been seen as a “game changer” because these cells provide access to human primary cells in any desired amount^4^. Accordingly, cells differentiated from hPSC lines have already been widely used for drug discovery^5,6^, including as a material for high-throughput screening, as well as for toxicology testing^5^. One of the most interesting features of those hPSC lines is that they provide the ability to select discrete cell phenotypes with a better relevance to the pathology under exploration. However, it remains to be demonstrated whether the results obtained using those newly developed, potentially more relevant *in vitro* models can be more predictive of subsequent clinical results than the results of previous cell models. To address this question, we focused on Friedreich ataxia (FRDA) based on our approach on the use of induced pluripotent stem cells (iPSCs) derived from patients’ fibroblasts. Indeed, two drugs, namely, nicotinamide and resveratrol, have been identified in classical cell models to promote the expression of frataxin^7,8^, which low level is responsible for the disease^9–12^. Nonetheless, resveratrol performed poorly in clinical trials in patients. Additionally, nicotinamide, even though it increased frataxin expression at high concentrations in peripheral blood mononuclear cells, had no effects at 8 weeks on patient’s neurological symptoms in an early phase open-label study^13,14^. One potential cause for this discrepancy was the fact that *in vitro* drug testing involved lymphoblastoid cells and fibroblasts, which may have been of little relevance for neurological disease. To explore this hypothesis, we compared the effects of the two drugs in *in vitro* cell models on frataxin expression at both RNA and protein levels in patients’ fibroblasts and in a presumed more relevant neuronal cell type derived from patients’ iPSCs cell lines.

## Results

### Generation of iPSCs, mesenchymal stem cells and neurons from FRDA patient’s fibroblasts

**Figure 1:**
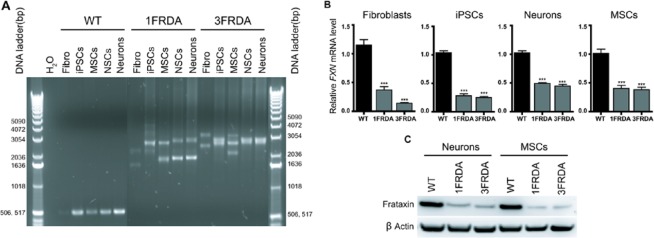
GAA triplet repeats and *FXN* expression in patient fibroblasts and their corresponding iPSC-derived MSCs, NSCs and neurons. (**A**) PCR analysis of *FXN* GAA repeats length in two distinct FRDA patient (1FRDA and 3FRDA) fibroblasts, iPSCs, MSCs, NSCs and neurons compared to those in wild-type (WT) cells. (**B**) Quantitative RT-PCR analysis of *FXN* transcript levels in 1FRDA and 3FRDA fibroblasts, iPSCs, neurons and MSCs relative to that in WT cells and normalized to 18S rRNA expression. Bars represent mean ± SEM (n = 3 independent experiments), ***p < 0.0001. (**C**) Western blot analysis of frataxin expression in WT cells, FRDA neurons and MSCs.

Primary fibroblasts from two FRDA patients (1FRDA and 3FRDA) were reprogrammed using the episomal vector-based strategy^15^. Both FRDA iPSC lines exhibited typical pluripotent cell morphology, expressed pluripotency markers (mRNA and protein), harboured a normal karyotype and were able to form *in vitro* embryoid bodies that expressed markers of the three embryonic germ layers (Supplementary Fig. 1A,B,E,F). Neither episomal integration nor the expression of the transgenes was detected by PCR analyses in the iPSCs (Supplementary Fig. 1C,D). According to established protocols, both FRDA iPSC lines were differentiated into homogeneous populations of either neurons or mesenchymal stem cells (MSCs) (Supplementary Fig. 1G,H). FRDA cells maintained GAA repeat expansions of a pathological size compared to the control (WT) cells (Fig. 1A). These expanded GAA repeats were different between cell types, as previously reported^16^, and caused a 60 to 80% decrease in *FXN* mRNA expression in FRDA cells compared to that in WT cells (Fig. 1B). Accordingly, downregulation of the protein frataxin was observed in FRDA iPSC-derived MSCs and neurons (Fig. 1C).

### Effects of nicotinamide and resveratrol treatment on frataxin expression depends on the cell types

The capacity of nicotinamide and resveratrol to modify the level of frataxin expression was evaluated in FRDA iPSC-derived MSCs and neurons. The respective parental fibroblasts were used as positive controls.

**Figure 2:**
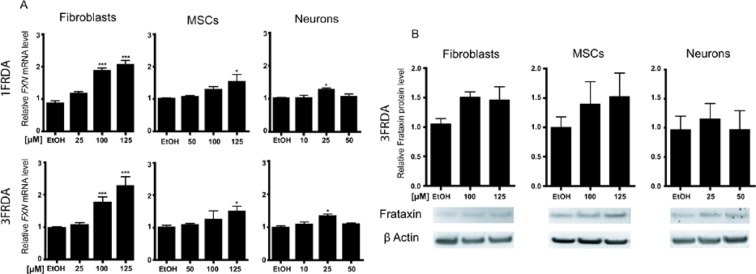
Comparison of *FXN* mRNA and protein expression in FRDA fibroblasts, MSCs and neurons treated with resveratrol. (**A**) Quantitative RT-PCR analysis of *FXN* transcript levels in 1FRDA and 3FRDA fibroblasts, MSCs and neurons treated with three doses of resveratrol. Data are expressed relative to that of EtOH-treated cells and normalized to 18S rRNA expression. Bars represent mean ± SEM (n = 3 independent experiments), *p < 0.05; ***p < 0.0001. (**B**) Western blot quantification of frataxin level in fibroblasts, MSCs and neurons under resveratrol treatment. Fibroblasts were exposed to resveratrol for 72 hours, while MSCs and neurons were treated for 48 hours. Frataxin levels were normalized to β-actin expression and are expressed relative to EtOH-treated cells. Bars indicate mean ± SEM (n = 3 independent experiments).

Quantitative RT-PCR analyses showed an ~2-fold increase in *FXN* mRNA expression in 1FRDA and 3FRDA fibroblasts treated with 100 µM resveratrol for 72 hours compared to the mRNA expression in mock-treated cells (Fig. 2A). This effect was even higher (2.3-fold) at 125 µM in both patients’ fibroblasts (Fig. 2A). In iPSC-differentiated MSCs, 125 µM resveratrol elicited a slight increase (1.6- and 1.5-fold for 1FRDA and 3FRDA, respectively) in *FXN* mRNA levels at 48 hours (Fig. 2A). Similar results were observed in 3FRDA MSCs after 72 hours (Supplementary Fig. 2A). In neurons, a different dose-response curve was observed. A very modest effect (1.3-fold upregulation) was detected with 25 µM resveratrol after 48 and 72 hours of treatment (Fig. 2A and Supplementary Fig. 2A). Higher concentrations were ineffective (data not shown). Similar results were obtained by normalizing *FXN* mRNA expression to the mean expression of a set of different housekeeping genes (Supplementary Fig. 2B). Consistent with the analysis of *FXN* mRNA, resveratrol did not induce a statistically significant change in frataxin expression (Fig. 2B).

**Figure 3:**
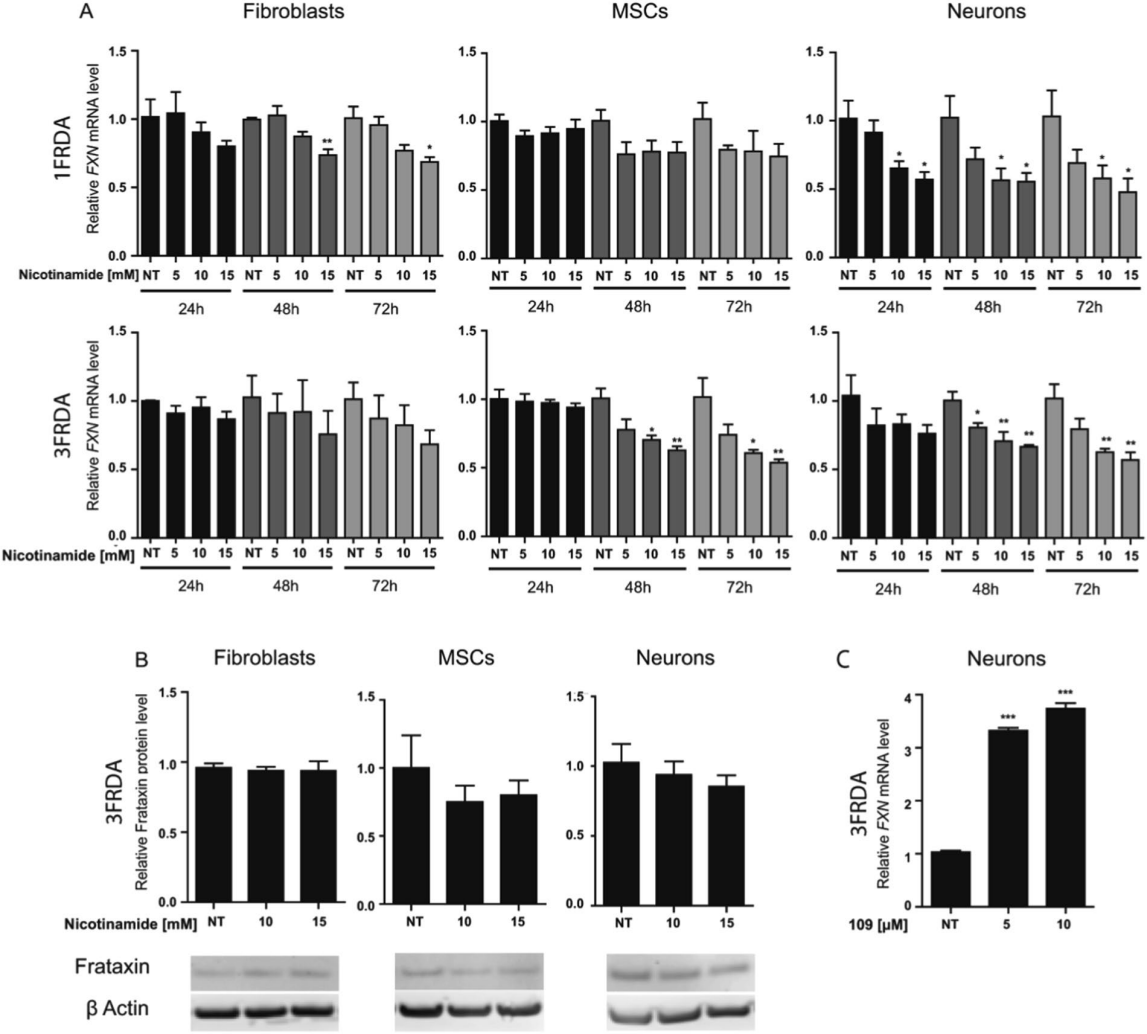
Effect of nicotinamide treatment on FXN expression in FRDA fibroblasts, MSCs and neurons. (**A**) Quantitative RT-PCR analysis of *FXN* transcript levels in 1FRDA and 3FRDA fibroblasts, MSCs and neurons treated with three repeated doses of nicotinamide for 24, 48 and 72 hours. Data are expressed relative to non-treated (NT) cells and were normalized to 18S rRNA expression. Bars represent mean ± SEM (n = 3 independent experiments), **p < 0.01; ***p < 0.0001. (**B**) Western blot quantification of frataxin expression levels in fibroblasts, MSCs and neurons under 72 hours of a daily repeated treatment with nicotinamide. Frataxin levels were normalized to β-actin and expressed relative to NT cells. Bars indicate mean ± SEM (n = 3 independent experiments). (**C**) Quantitative RT-PCR analysis of *FXN* transcript levels in 3FRDA neurons treated with 5 µM or 10 µM of HDAC inhibitor 109 for 24 hours. Data are expressed relative to non-treated cells and normalized to 18S rRNA expression. Bars indicate mean ± SEM (n = 2 independent experiments including 6 technical replicates for each), ***p < 0.0001.

Nicotinamide treatments were performed at 5, 10 and 15 mM for 24, 48 and 72 hours in fibroblasts and iPSC-derived MSCs and neurons (Fig. 3). Healthy (GM14926) and patient-derived (GM15850 and GM16234) EBV transformed lymphoblastoid cell lines were treated with 10 mM nicotinamide for 16 hours as previously described^7^ (Supplementary Fig. 4B). According to Chan *et al*., we observed a slight increase in the *FXN* mRNA levels in the lymphoblastoid cell lines. However, nicotinamide resulted in a dose- and time-dependent decrease in the expression of *FXN* mRNA in fibroblasts and iPSC derivatives (Fig. 3A). Similar results for MSCs and neurons treated with nicotinamide for 72 hours were obtained by normalizing *FXN* mRNA expression to the mean expression of a set of different housekeeping genes (Supplementary Fig. 4A). This outcome on *FXN* expression was not associated with a toxic effect from nicotinamide (Supplementary Fig. 3). At the protein level, there was no significant difference in frataxin expression between treated and non-treated cells (Fig. 3B). In order to assess nicotinamide efficiency, we quantified by western blot the level of acetylation on histone H3 lysine 9 (H3K9 Ac) in 3FRDA neurons treated with 10 and 15 mM nicotinamide for 72 hours (Supplementary Fig. 4C). This experiment showed that nicotinamide treatment increased histone H3K9 acetylation level in 3FRDA neurons compared to non-treated cells (NT).We also treated 3FRDA iPSC-neurons with 5 µM or 10 µM RG2833 (HDAC inhibitor 109) and observed a significant upregulation in *FXN* mRNA expression similar to that observed by Soragni *et al*.^17^ (Fig. 3C).

## Discussion

In the context of Friedreich ataxia, this study has evaluated the use of presumably more relevant cell types derived from patients’ iPSCs as predictive tools for two compounds, namely, resveratrol and nicotinamide, that were recently revealed to have a poor efficacy in patients. Whereas these compounds were initially selected for clinical investigation due to their beneficial effect *in vitro* on frataxin expression in fibroblast and lymphoblastoid cells, our results indicated that these molecules failed to do so in two FRDA iPSC line-derived neurons. By comparing the effects of resveratrol and nicotinamide on different cell types that may be considered either as non-relevant to the disease, such as patients’ fibroblasts, or more relevant, such as neurons, the present study also highlighted a differential response to these compounds, suggesting that iPSC-based assays may be a useful addition to the early steps of drug discovery.

Because of the gap between the results obtained for drug testing in *in vitro* cellular models and the drug efficacy in patients, the quest for more predictive *in vitro* systems is considered an urgent challenge of modern drug discovery^1^. The development of more effective treatments for neurological diseases has been especially impeded by the difficulties in accessing primary samples of human disease-relevant cells. FDRA is an example of that situation. This autosomal recessive disease is the most common form of inherited ataxias, typically resulting from an expanded GAA trinucleotide repeat in the first intron of the *FXN* gene that leads to the reduced expression of frataxin^18–20^. Therapeutic approaches have largely focused on the identification of chemical compounds that would increase frataxin levels. However, success has been elusive in clinical trials^11,21,22^. Nicotinamide, which increased *FXN* mRNA expression in patients’ lymphoblasts and in an *FXN*-transgenic mouse^7^, did not show significant improvements in neurological measure after 8 weeks of treatment during an open-label phase I trial, even though the frataxin levels were slightly increased in the patients’ blood^13^. In the same vein, resveratrol was recently tested in a non-randomized phase I/II clinical study based on previous observations showing a 1.5- to 2-fold increase in frataxin expression in patients’ lymphoblasts and fibroblasts and a 1.5- fold increase in human frataxin in the brain of a humanized mouse model of the disease^8^. Patients treated with a high dose of resveratrol were observed to have a slight improvement in their neurological functions, but that improvement appeared in the absence of an effect on frataxin expression, as was recorded in the blood^14^. Although long-term assessments of the neurological benefits have not been conducted for either drug, the results of those clinical trials obtained so far did not match the expectation derived from the *in vitro* drug testing on which they were based. Conversely, the rather negative results of the *in vitro* cell testing based on iPSC-derived cells could be considered as a warning had they been available at the time the clinical trials were initiated.

Our results also support the notion that there may be very differential responses as a function of the assayed cell type. This seems to mirror the differential expression of the disease that affects different organs and tissues despite a ubiquitous *FXN* gene anomaly. Accordingly, Soragni *et al*. recently described a differential response to another class of HDAC inhibitors (compound 109) in patient-derived fibroblasts compared to the response in FRDA iPSC-derived neurons^17^. Moreover, a number of published studies involving the generation of FRDA iPSC-derived neurons and cardiomyocytes have described cellular phenotypes^23–25^ and showed that the HDAC inhibitor compound 109 can partially rescue some of these phenotypes^24,25^. Now that the availability of patient-derived iPSC lines allows it, these results suggest that it may be of interest to systematically assess the efficacy of potential therapeutic compounds on human cell types that are as relevant as possible to the ones that are affected by a disease. Our results support the hypothesis that those cells are a more predictive model in the context of *in vitro* drug discovery and may thus enable better informed decisions in the translation of experimental pharmacological results to the clinic.

## Material And Methods

### Fibroblast reprogramming and pluripotent stem cell culture

The fibroblasts used in this study were isolated from patient biopsies performed in the Assistance Publique Hôpitaux de Paris for FRDA patients and provided by Aurélien Bayot from Pierre Rustin’s team^26^. Informed consent was obtained from patients and/or legal guardians according to the protocols approved by the Robert Debré Hospital ethical committee (Paris, France). All experiments were performed in accordance with relevant guidelines and regulations. Fibroblasts were cultured in a medium consisting of DMEM, high glucose, and GlutaMAX Supplement supplemented with 10% foetal bovine serum (Sigma), 1% non-essential amino acids and 1 mM sodium pyruvate (Life Technologies). The human iPSC control line (WT, i90c16) was derived from IMR-90 lung fibroblast cells (ATCC® CCL-186). All iPSCs were obtained as previously described by Yu *et al*. using Addgene plasmids 20925, 20926 and 20927. Molecular characterization of the pluripotency and self-renewal capacities of these cells was performed as described previously^15^.

Human ES cells (H9, passages 40–60; WiCell Research Institute), WT and FRDA iPSCs (passages 25–40) were maintained on a layer of mitotically inactivated mouse embryonic fibroblasts (MEFs, Globalstem). Human PSCs were cultured in DMEM/F12 GlutaMAX supplemented with 20% knockout serum replacement, 1 mM non-essential amino acids, 0.55 mM β-mercaptoethanol, and 10 ng/ml recombinant human FGF2 (hESC medium) (all from Life Technologies). Culture medium was changed daily and the cells were manually passaged every 5–7 days.

### Pluripotent stem cell differentiation

For embryoid body (EB) differentiation, iPSC colonies growing on MEFs were detached with 1 mg/ml collagenase for 10 min at 37 °C, resuspended in hESC medium without FGF2 and cultured in low-attachment 6-well plates for 7 days. The EBs were then plated on 0.1% gelatine-coated plates and maintained for another 7 days prior to immunostaining.

NSC differentiation was performed as described in^27^. Neuronal differentiation was induced by plating the NSCs at a low density (50 000 cells cm^2^) in poly-ornithine/laminin-treated culture plates in N2B27 medium consisting of DMEM/F12, neurobasal, N2 and B27 supplement, 50 μM β-mercaptoethanol and penicillin/streptomycin (Life Technologies) without FGF2 and EGF. The medium was changed every 4 days, and neurons were obtained after 18-21 days of terminal differentiation.

MSCs were obtained using an adaptation of a previously described protocol^28^ as published in^27^. For all subsequent experiments, MSCs were thawed and cultured on 0.1% gelatine‐coated dishes in MSC medium without FGF2 and Aa2‐P.

### Cell culture and drug treatments

Fibroblasts and MSCs were seeded in 0.1% gelatine‐coated 6-well plates at a density of 15 000 cells/cm^2^ and treated with nicotinamide or resveratrol 24 hours after seeding. Nicotinamide (Sigma-Aldrich) treatments were performed every day with various drug concentrations (5 mM, 10 mM and 15 mM), and cells were collected at 24 hours, 48 hours and 72 hours. EBV-transformed lymphoblastoid cells (GM14926, GM14664, GM15850, GM15851, GM16234, and GM16798) were purchased from the National Institute of General Medical Sciences Human Genetic Cell Repository at the Coriell Institute, Camden, NJ, USA. These cells were cultured and treated with nicotinamide as described by Chan *et al*.^7^. For resveratrol (Sigma-Aldrich), cells were treated once with concentrations ranging from 25 µM to 125 µM for fibroblasts and MSCs and from 10 µM to 50 µM for neurons. EtOH 0.1% was used as a control treatment for resveratrol. Cells were collected at 48 hours and 72 hours. For the HDAC inhibitor RG2833 (RGFP109, Selleckchem), 3FRDA iPSC-neurons were treated for 24 hours with 5 µM or 10 µM. Drug concentrations and duration of the treatments were defined based on the literature^7,8,17^.

### Determination of GAA repeats length

GAA repeats at the *FXN* locus were amplified using the primers GAA-104F and GAA-629R as described previously^16^.

### TaqMan array gene profiling

TaqMan microfluidic cards (Applied Biosystems) were processed as described by the manufacturer’s instructions. In brief, 100 ng of cDNA was mixed with TaqMan Universal PCR Master Mix (Applied Biosystems) before being injected into the microfluidic cards and dispersed into the wells by centrifugation. Microfluidic cards were sealed, and qPCR assays were run on the 7900HT Fast Real-Time System (Life Technologies) using ABI PRISM 7900 Sequence Detection System software (v2.4; Applied Biosystems, Life Technologies). The cycle thresholds were analysed using DataAssist Software (v2.0). Hierarchical clustering of gene expression and heatmap representation was performed with dChip software (Harvard).

### mFISH karyotype analysis

Cells were conditioned with colchicine (Eurobio) for 90 min, warmed with a hypotonic solution (5 mg/mL KCL) and fixed with Carnoy’s fixative. mFISH 24Xcite probe (Metasystem) and ProLong Gold Antifade Mountant with DAPI (Life Technologies) were used for mFISH staining. Then, 30–70 metaphases were acquired with Metafer MetaSystems software coupled to an AxioImager Zeiss Z2 microscope equipped with a camera cool cube and 10X and 63X objectives. Images were analysed with Isis software (MetaSystems).

### Episomal integration and expression detection

RT-PCR analysis of transgene expression and PCR analysis of episomal vectors integration were carried out as previously described with the same primers^15^. The cDNA from FRDA fibroblasts transfected with a combination of the 3 episomal vectors (day 4 post-transfection) was used as a positive control. Episomal DNA was used as a positive control for PCR analysis.

### mRNA purification and qRT-PCR

Total RNA was isolated using the RNeasy Mini or Micro Plus Extraction Kit and QIAcube instrument (QIAGEN, Courtaboeuf, France) according to the manufacturer’s protocol. The RNA level and quality were checked using a NanoDrop Spectrophotometer. cDNA was synthesized from 500 ng of RNA with SuperScript III Reverse Transcriptase (Life Technologies) using random primers. Quantitative PCRs were performed with Power SYBR Green PCR Mix using an ABI 7900 system (Applied Biosystems). Quantification of gene expression was based on the 2^−ΔΔCt^ method and normalized to 18S rRNA expression or to the mean of the Ct values of a set of housekeeping genes including GAPDH, HPRT1 and PPIA. qRT-PCR primers FRAT2-fwd and FRAT2-rev were used for *FXN* gene expression as described previously^26^.

### Immunocytochemistry

Cells were fixed in 4% PFA and incubated overnight at 4 °C with primary antibodies. Secondary antibodies and a DAPI counterstain were applied for 1 hour. Antibodies are listed in Supplemental Table 1. Image acquisitions were performed on an epifluorescence microscope (Imager Z1; Carl Zeiss, LePecq, France) using the AxioVision image capture equipment and software.

### Flow cytometry analysis

Cells were detached from culture plates using trypsin 0.05% EDTA (Life Technologies). Immunostaining was performed on 100 000 cells per experiment at 4 °C for 30 min. The antibodies used are described in Supplemental Table 1. Cells were then incubated with 10 µg/ml DAPI solution. Analysis was performed on a MACSQuant flow cytometer (Miltenyi Biotec) using FlowJo software (Tree Star, Inc.). A total of 15 000 events were recorded for each sample analysed.

### Western immunoblotting

Proteins from whole-cell lysates were collected using RIPA buffer supplemented with a 1% protease inhibitor cocktail (Sigma-Aldrich) and 10% phosphatase inhibitors (Roche). The proteins were then separated on 4–12% Nu-PAGE® Bis-Tris gels and transferred onto nitrocellulose membranes using the iBlot® Gel Transfer Device (all from Life Technologies). For frataxin detection, membranes were blocked in PBS containing 0.1% Tween-20 and 5% non-fat dry milk and then incubated overnight at 4 °C with frataxin primary antibody. Blots were stained with HRP-conjugated secondary antibodies and visualized using the enhanced chemiluminescence reagent ECL plus (Amersham Pharmacia Biotech). Image acquisition was performed using the ImageQuant CDD camera (GE Healthcare, Saclay, France), and densitometric analysis was carried out using Fiji software. For the detection of acetylated histone 3, membranes were blocked in Odyssey blocking buffer (Li-Cor) and then incubated with primary antibodies (acetyl-Histone H3 (Lys9) or histone H3, pan) diluted at 4 °C overnight. Membranes were then incubated with secondary antibodies and proteins were detected by fluorescence (Odyssey, Li-Cor) and quantified following the manufacturer’s instructions. The antibodies used are described in Supplemental Table 1.

### Viability, cytotoxicity, and apoptosis assay

MSCs from 3FRDA were seeded at 5 000 cells per well in 96-well plates and were treated with a dose range of nicotinamide for 72 hours or a dose range of ionomycin and staurosporine for 24 hours. Viability, cytotoxicity, and apoptosis events were assessed using the ApoTox-Glo Triplex Assay (Promega). Briefly, after incubation of cells with the “Viability/Cytotoxicity Reagent” for 60 min at 37 °C, the resulting cell viability and cytotoxicity fluorescence were measured respectively at 400 nm excitation/505 nm emission and 485 nm excitation/520 nm emission using the CLARIOstar microplate reader (BMG LABTECH). Cells were then incubated with the “Caspase-Glo 3/7 reagent” for 60 min at room temperature, and caspase activation was determined with luminescence measurements using the CLARIOstar microplate reader (BMG LABTECH).

### Statistical analysis

Statistical analysis was performed by one-way analysis of variance (ANOVA) using Dunnett’s comparison test. Values of p < 0.05 were considered significant (*p < 0.05, **p < 0.01, ***p < 0.001).

## Supplementary information

supplementary informations

## Data Availability

Data generated during the current study are available from the corresponding author upon reasonable request.
