# Supplementary material for: Induced pluripotent stem cells-derived neurons from patients with Friedreich ataxia exhibit differential sensitivity to resveratrol and nicotinamide
Source: Sci Rep. 2019 Oct 10;9:14568. doi: 10.1038/s41598-019-49870-y (PMC6787055; doi:10.1038/s41598-019-49870-y)
Supplement: Supplementary file 1 — supplementary informations [file 41598_2019_49870_MOESM1_ESM.pdf]

Supplementary Information

Induced pluripotent stem cells-derived neurons from patients with Friedreich ataxia exhibit differential sensitivity to resveratrol and nicotinamide

Pauline Georges, Maria-Gabriela Boza-Moran, Jacqueline Gide, Georges Arielle Pêche, Benjamin Forêt, Aurélien Bayot, Pierre Rustin, Marc Peschanski, Cécile Martinat, Laetitia Aubry

Supplemental Figure 1

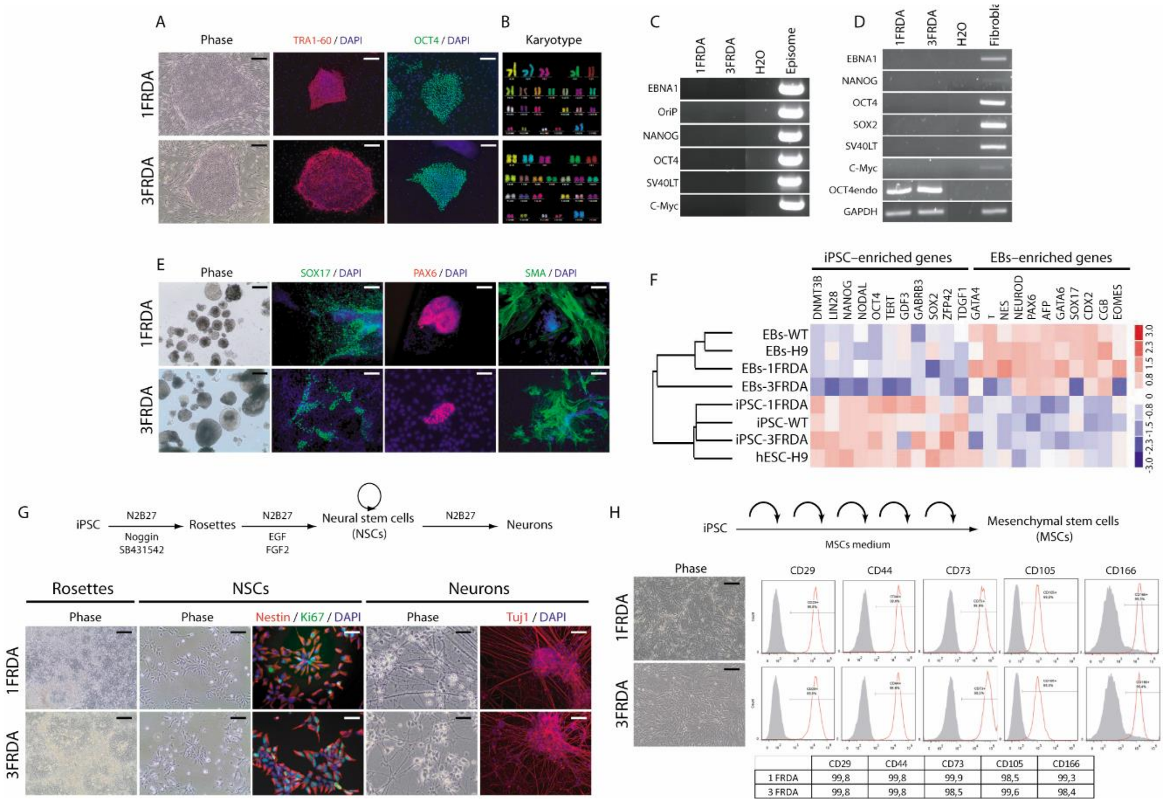

	CD29	CD44	CD73	CD105	CD166
1 FRDA	99,8	99,8	99,9	98,5	99,3
3 FRDA	99,8	99,8	98,5	99,6	98,4

Supplemental Figure 1. Establishment and characterization of two distinct FRDA patients-derived human-induced pluripotent stem cells and their derivatives. (A) Representative phase-contrast image of i90c16-iPSCs colonies on feeder cells and immunostaining showing the expression of the pluripotency markers TRA1-60 and OCT4. (B) mFISH chromosome analysis of FRDA-iPSCs showing a normal caryotype (46, XX). (C) PCR analysis of episomal DNA integration using FRDA-iPSCs genomic DNA and primers specifically targeting OCT4, SV40LT, C-MYC, NANOG, OriP and EBNA1 from the episomal vectors. Episomal vectors (Episome) and GAPDH were used as controls. (D) RT-PCR analysis of transgene expression using FRDA-iPSCs mRNA and primers specifically targeting OCT4, C-MYC, EBNA1, SOX2, SV40LT, NANOG from the episomal vectors and endogenous OCT4. Fibroblasts transfected with the 3 episomal vectors (4 days after transfection) were used as positive control. (E) *In vitro* embryoid body formation from FRDA-iPSCs showing three germ layer differentiation as illustrated by the presence of endodermal SOX17+ cells, neuroectodermal PAX6+ cells and mesodermal SMA+ cells. (F) Unsupervised hierarchical clustering of genes expression that are differentially expressed between human iPSCs and embryoid bodies (EBs). (Left) Eleven well-known human pluripotent stem cell-enriched genes; (right) eleven three germ layers-enriched genes. The color key is shown on the right side. The H9 hES cells line was used as a reference. (G) Schematic representation and phenotypical characterization of neuronal FRDA-iPSCs differentiation using the dual SMAD inhibition method (Noggin and SB431542). Immunostaining showed the expression of the neural (Nestin) and proliferating (Ki67) markers in NSCs and the expression of the neuronal markers TUJ1 in neurons. (H) Schematic illustration of MSCs differentiation protocol, phase-contrast image of FRDA MSCs, and flow cytometry analysis of MSCs markers (CD29, CD44, CD73, CD105 and CD166) expression in FRDA MSCs. Scale bars = 50 µm.

Supplemental Figure 2

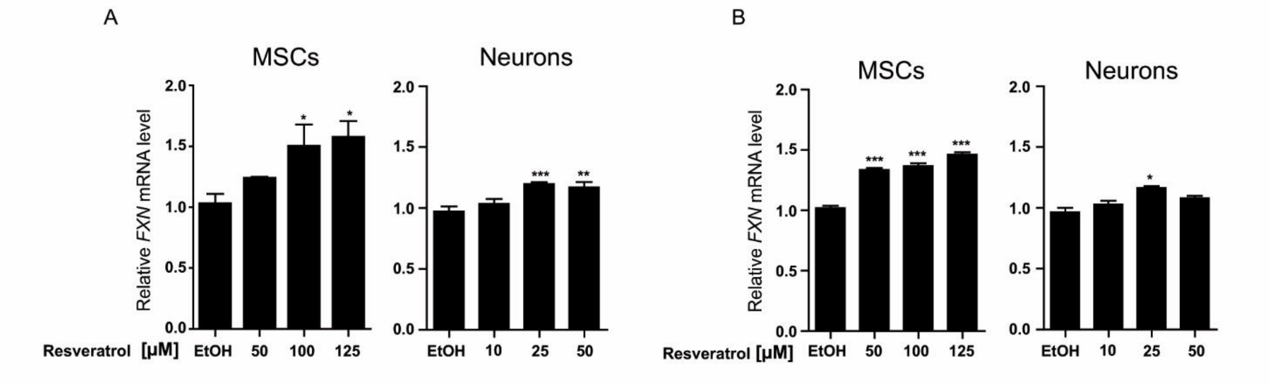

Supplemental Figure 2. Effect of resveratrol treatment on *FXN* mRNA level in 3FRDA iPSC-derived MSCs and neurons. (A) Quantitative RT-PCR analysis of *FXN* transcript levels in 3FRDA MSCs and neurons treated with different doses of resveratrol for 72 hours. Data are expressed relative to EtOH and normalized to 18S rRNA expression (n = 3 independent experiments). (B) Quantitative RT-PCR analysis of *FXN* transcript levels in 3FRDA MSCs and neurons treated with different doses of resveratrol for 48 hours. Data are expressed relative to EtOH and normalized to the mean expression of a set of housekeeping genes including PPIA, GAPDH and 18s rRNA (n = 2 independent experiments). Bars represent mean ± SEM, *p<0.05; ** p<0.01; ***p<0.001.

Supplemental Figure 3

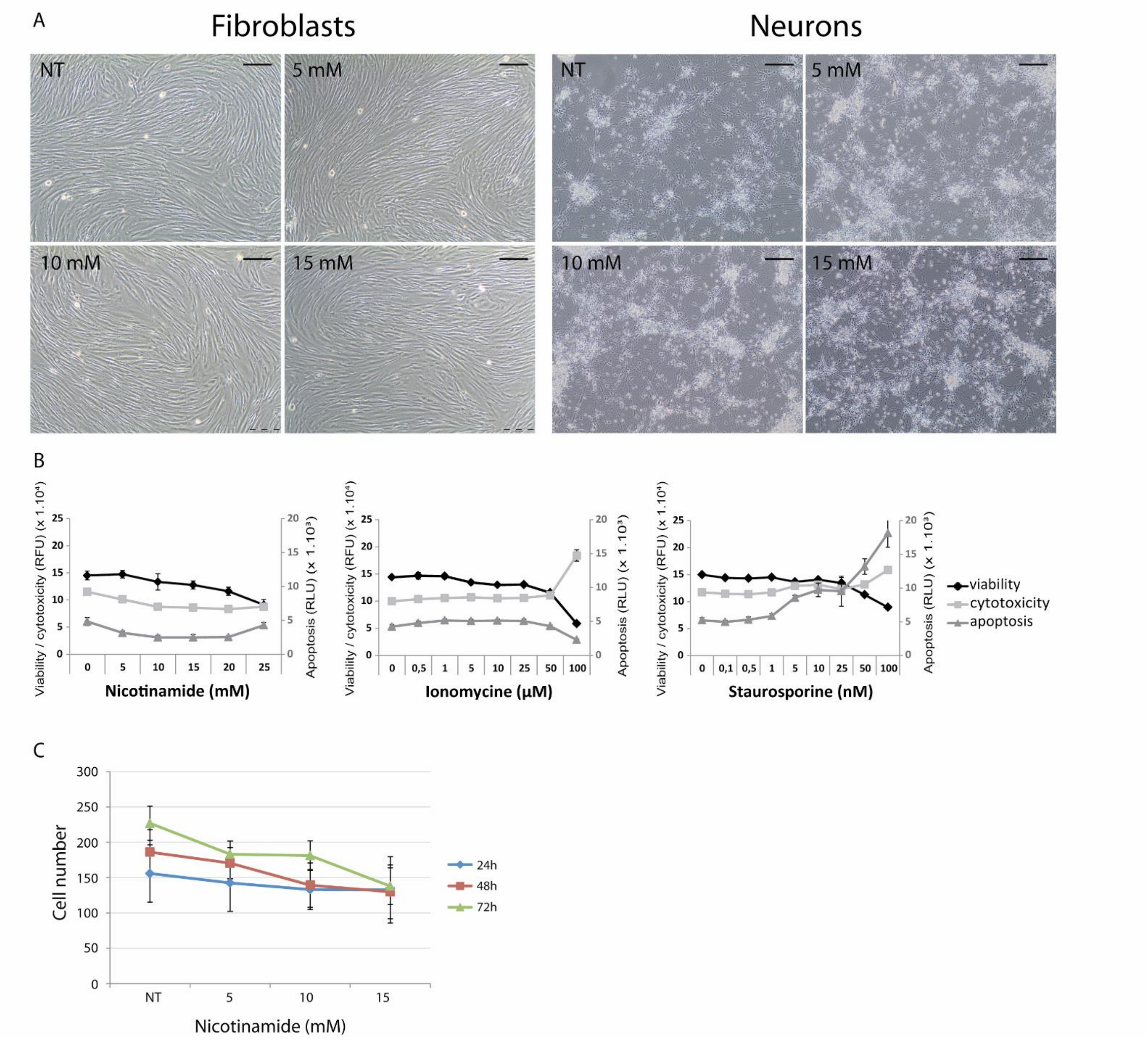

Supplemental Figure 3. Assessment of nicotinamide treatment toxicity. (A) Phase contrast images of 3FRDA fibroblasts and neurons treated with three daily repeated doses (5 mM, 10 mM and 15 mM) of nicotinamide for 72 hours. (B) 3FRDA MSCs were incubated with vehicle or increasing concentrations of nicotinamide for 72 hours or staurosporine and ionomycin for 24 hours before measurement of viability, apoptosis, and toxicity with the ApoTox kit. (C) Nicotinamide toxicity assessment in 3FRDA MSCs treated with daily repeated doses for 24, 48 and 72 hours. Cell nuclei were quantified by high content imaging using Hoechst staining. Results of residual nuclei number are expressed as means ± SEM. RFU, relative fluorescence unit; RLU, relative luminescence unit. Scale bars = 50 µm.

Supplemental Figure 4

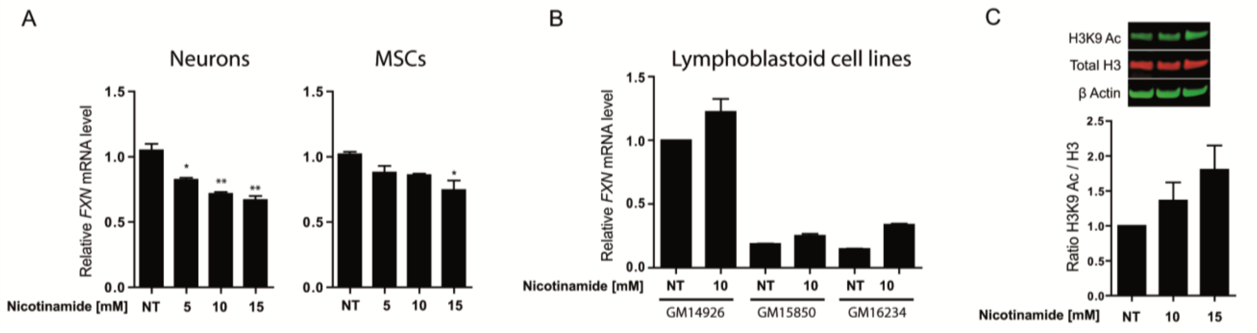

Supplemental Figure 4. Effect of nicotinamide treatment on *FXN* expression in neurons, MSCs and EBV-transformed lymphoblastoïd cell lines and on the level of histone 3 acetylation in neurons. (A) Quantitative RT-PCR analysis of *FXN* transcript levels in 3FRDA neurons treated with nicotinamide for 72 hours. Data are expressed relative to non-treated cells and normalized to the mean expression of a set of housekeeping genes including HPRT1, PPIA, GAPDH and 18s rRNA. Bars indicate mean ± SEM, *p<0.05, ** p<0.01. (B) Quantitative RT-PCR analysis of *FXN* transcript levels in healthy (GM14926) and in patient derived (GM15850 and GM16234) EBV-transformed lymphoblastoïd cell lines treated with 10 mM nicotinamide for 16 hours. Data are expressed relative to non-treated GM14926 cells and normalized to 18S rRNA expression. (C) Western blot quantification of the level of histone 3 acetylation at lysine 9 (H3K9 Ac) in 3FRDA neurons under nicotinamide treatment. Neurons were exposed to nicotinamide for 72 hours. H3K9 Ac levels were normalized to total H3 and expressed relative to non-treated cells (NT).

Supplemental Figure 5

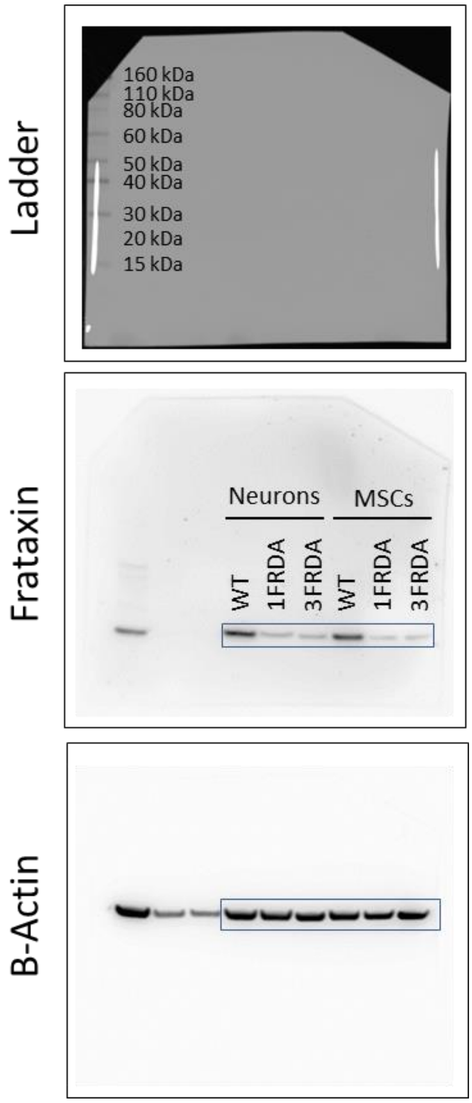

Supplemental Figure 5. Frataxin expression in WT and FRDA iPSC-derived neurons and MSCs. Uncropped western blot image of frataxin and β-actin expression in WT, 1FRDA and 3FRDA iPSC-derived MSCs and neurons (related to figure 1C).

Supplemental Figure 6

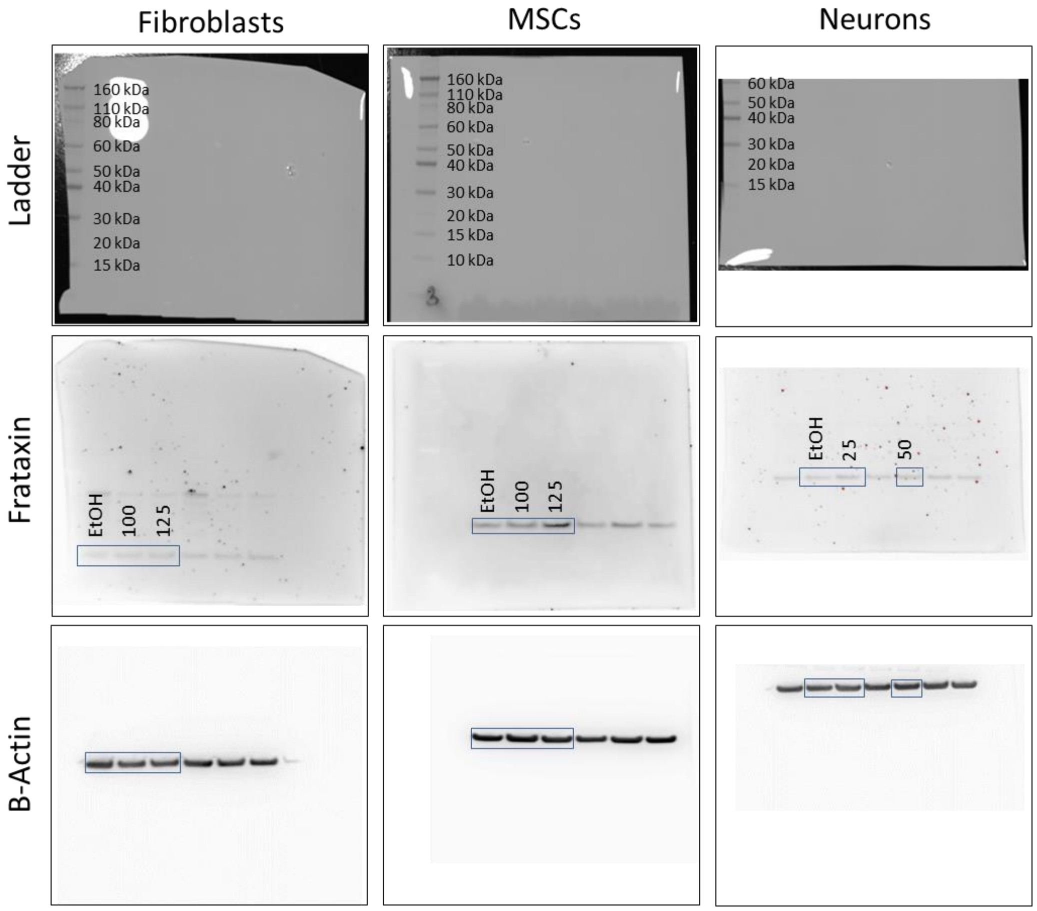

Supplemental Figure 6. Effect of resveratrol treatment on frataxin expression in 3FRDA fibroblasts, MSCs and neurons. Uncropped western blot images of frataxin and β-actin expression in 3FRDA fibroblasts, iPSC-derived MSCs and neurons treated with vehicle control ethanol (EtOH) or resveratrol at 100 µM and 125 µM for fibroblasts and MSCs, or 25 µM and 50 µM for neurons. Fibroblasts were exposed to resveratrol for 72 hours, while MSCs and neurons were treated for 48 hours (related to figure 2B).

Supplemental Figure 7

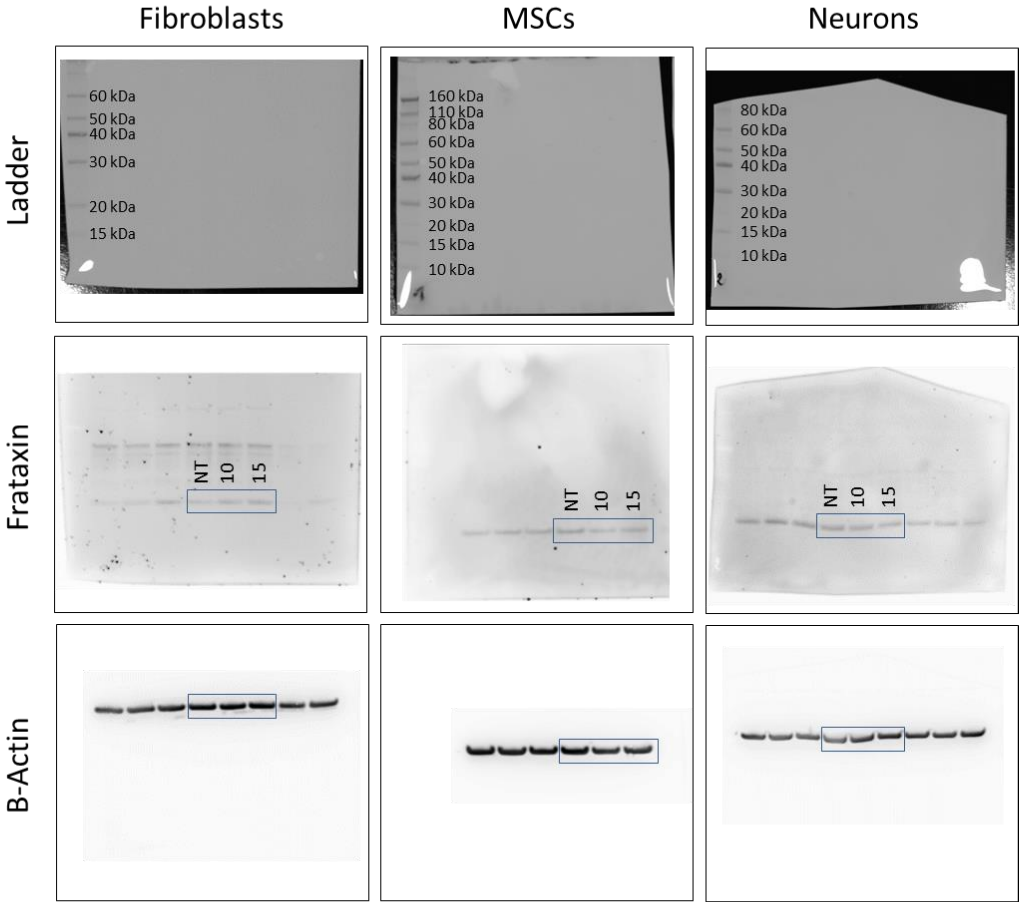

Supplemental Figure 7. Effect of nicotinamide treatment on frataxin expression in 3FRDA fibroblasts, MSCs and neurons. Uncropped western blot images of frataxin and β-actin expression in 3FRDA fibroblasts, iPSC-derived MSCs and neurons treated for 72 hours with 10 mM or 15 mM nicotinamide, compared to non-treated cells (NT) (related to figure 3B).

Supplemental Table 1. List of antibodies, providers and dilutions.

Application	Anitbody	Host	Provider	Dilution
Immunicytochemistry	OCT4	Rabbit	Cell signaling Technology	1/500
	TRA-1-60	Mouse	Cell signaling Technology	1/500
	Nestin	Mouse	Merck Millipore	1/1000
	Ki67	Mouse	Agilent Dako	1/500
	Tuj-1 (beta-III tubulin)	Mouse	Eurogentec	1/1000
	PAX6	Rabbit	Covance	1/500
	SOX17	Goat	R&D systems	1/200
	SMA	Mouse	Agilent Dako,	1/100
FACS	CD29 FITC	Mouse	BioLegend	1/20
	CD44 APC-H7	Mouse	BD biosciences	1/20
	CD73 APC	Mouse	BioLegend	1/40
	CD105 PE/Cy7	Mouse	BioLegend	1/20
	CD166 PE	Mouse	BioLegend	1/10
Western Blot	Frataxin	Mouse	abcam	1/500
	Acetyl-Histone H3 (Lys9)	Rabbit	Cell Signaling Technology	1/1000
	Histone H3, pan	Rabbit	Merck Millipore	1/1000
	β-Actin−Peroxidase	Mouse	Sigma	1/50000
	β-Actin	Mouse	LI-COR	1/2000
